# Comparative analysis of nine *Tilletia indica* genomes for the development of novel microsatellite markers for genetic diversity and population structure analysis

**DOI:** 10.3389/fmicb.2023.1227750

**Published:** 2023-07-13

**Authors:** Prem Lal Kashyap, Sudheer Kumar, Ravi Shekhar Kumar, Anju Sharma, Annie Khanna, Kajal, Shubham Raj, Poonam Jasrotia, Gyanendra Singh

**Affiliations:** ICAR-Indian Institute of Wheat and Barley Research (IIWBR), Karnal, India

**Keywords:** aggressiveness, genome, Karnal bunt, microsatellite, population structure, structure, *Tilletia indica*

## Abstract

Karnal bunt (KB; *Tilletia indica*) is the prime quarantine concern for quality wheat production throughout the world. The most effective approach to dealing with this biotic stress is to breed KB-resistant wheat varieties, which warrants a better understanding of *T. indica* genome architecture. In India, the North Western Plain Zone is the prime hot spot for KB disease, but only limited efforts have been made to decipher *T. indica* diversity at the genomic level. Microsatellites offer a powerful and robust typing system for the characterization and genetic diversity assessment of plant pathogens. At present, inadequate information is available with respect to the development of genome-derived markers for revealing genetic variability in *T. indica* populations. In current research, nine complete genome sequences of *T. indica* (PSWKBGH_1, PSWKBGH_2, PSWKBGD_1_3, RAKB_UP_1, TiK_1, Tik, DAOMC236408, DAOMC236414, and DAOMC236416) that exist in the public domain were explored to know the dynamic distribution of microsatellites. Comparative genome analysis revealed a high level of relative abundance and relative density of microsatellites in the PSWKBGH_1 genome in contrast to other genomes. No significant correlation between microsatellite distribution for GC content and genome size was established. All the genomes showed the dominance of tri-nucleotide motifs, followed by mono-, di-, tetra-, hexa-, and penta-nucleotide motifs. Out of 50 tested markers, 36 showed successful amplification in *T. indica* isolates and produced 52 different alleles. A PCR assay along with analysis of the polymorphic information content (PIC) revealed 10 markers as neutral and polymorphic loci (PIC 0.37). The identified polymorphic SSR loci grouped a geographically distinct *T. indica* population of 50 isolates representing seven Indian regions (Jammu, Himachal Pradesh, Punjab, Haryana, Uttarakhand, Uttar Pradesh, and Rajasthan) into four distinct clusters. The results of the analysis of molecular variance identified 94% genetic variation within the population and 6% among the population. Structure analysis also confirmed the existence of four genetically diverse groups containing admixtures of *T. indica* isolates across populations. In nutshell, the current study was successful in identifying novel, neutral and polymorphic microsatellite markers that will be valuable in offering deep insight into the evolutionary relationship and dynamics of the *T. indica* population for devising effective KB management strategies in wheat.

## Introduction

*Tilletia indica,* which causes Karnal bunt (KB) disease, is an important quarantine fungus that negatively influences the quality of wheat produce throughout the globe (Emebiri et al., 2021). The pathogen was first recorded in April 1931 from Karnal town in India (Mitra, 1931) and later reported from different countries, including the United States, Brazil, Pakistan, Mexico, Nepal, South Africa, Afghanistan, Syria, and Iran (Duhan et al., 2022). At present, more than 86 countries have banned wheat imports by executing strong quarantine policies and following a zero tolerance policy on wheat trade from KB-affected countries (Rush et al., 2005; Sansford et al., 2008; Bishnoi et al., 2020; Gurjar et al., 2021). In India, KB has been observed regularly in the North-Western plains and Tarai region of Himachal Pradesh, Jammu, Punjab, Uttrakhand, and Uttar Pradesh regions of India (Parveen et al., 2015; Kashyap et al., 2022). *T. indica* is a soil-, seed-, and air-borne fungus and has the potential to reside for several years in soil, wheat straw, and farmyard manure (Kashyap et al., 2011). After wheat sowing, *T. indica* fungus enters the seed via the germinal point and produces a brownish-black mass of teliospores with a decaying fish-like smell by producing trimethylamine (Kumar et al., 2004). Further, infected wheat seeds showed partial colonization and resulted in bunted grain (Riccioni et al., 2008; Bala et al., 2022). It has been noticed that the deterioration in the quality of wheat grain varies with the severity of *T. indica* infection during the spike growth stage (boot leaf stage or Zadok’s stage 49) in wheat (Kaur and Kaur, 2005; Goates and Jackson, 2006). Kashyap et al. (2018) documented that more than 3% KB infection in wheat grains had a negative impact on the appearance and taste of chapattis, cookies, and bread. However, >5% infection in wheat grains was unsuitable as a food source for humans (Sekhon et al., 1980; Warham, 1986; Ullah et al., 2012; Kumar et al., 2017; Kashyap et al., 2019a). Published literature also indicated that the wheat export from KB-affected areas resulted in production losses of 0.2–0.5% (Sharma et al., 2022). However, losses up to 40% have been observed in those areas where KB-susceptible varieties were grown by farmers (Bishnoi et al., 2021). In India, Shakoor et al. (2015) documented a yield loss due to KB of nearly 0.5%. However, yield losses up to 1% have been reported from Mexico (Iquebal et al., 2021). Unfortunately, effective and timely control of KB has become a difficult task because of several factors, including *T. indica* dispersal mode, the non-availability of KB-resistant wheat cultivars, and the survival of *T. indica* spores in the soil for several years (Parveen et al., 2013). Further, cultural and fungicide-based management strategies are not offering desirable results in managing KB because of the heterothallic nature and sporadic occurrence of *T. indica* (Parveen et al., 2015; Kashyap et al., 2019a). Besides this, hybridization in *T. indica* spores also stimulates recombination and further helps in the rising genetic diversity spectrum of the fungus (Singh and Gogoi, 2011). In such a situation, virulence data alone is inadequate to offer a suitable and better insight into the existing diversity in the field population of *T. indica*. Hence, a deep and comprehensive understanding of genetic diversity at the genomic scale becomes obligatory for efficient utilization of resistance sources and to discover new changes in the pathotype distribution or structure of the evolving *T. indica* population.

Over the past several decades, a series of traditional approaches, for instance, cultural distinctiveness, phenology, virulence, monosporidia, wheat-*T. indica* interaction at physiological and biochemical levels, etc., have been explored for resolving the mystery related to *T. indica* variability (Bonde et al., 1996; Pannu and Chahal, 2000; Sharma et al., 2002; Kumar et al., 2004; Thirumalaisamy and Singh, 2012; Gupta et al., 2015, 2017; Pandey et al., 2018, 2019; Kashyap et al., 2020). However, the laborious, time-consuming, and environmentally prejudiced nature of the aforementioned methods is their prime demerits. These methods also lack precision and accuracy. There are a flood of research reports that illustrate the potential of nucleic acid-derived markers for unzipping the variation among fungal pathogens at the genome level (Kumar et al., 2013; Kashyap et al., 2015,2019b; Goswami et al., 2017; Choudhary et al., 2018; Jiménez-Becerril et al., 2018; Prasad et al., 2018). For instance, genetic markers such as inter simple sequence repeats (ISSR) and random amplified polymorphic DNA (RAPD) have been extensively utilized to understand the genetic diversity of *T. indica* isolates (Avinash et al., 2000; Seneviratne et al., 2009; Aggarwal et al., 2010; Parveen et al., 2015; Aasma et al., 2022). Unfortunately, the aforementioned markers are dominant and unable to determine analogous reproducibility across populations, thereby being of little significance, especially for comparative genotyping studies (Agarwal et al., 2008; Rao et al., 2018). Alternatively, microsatellites [Syn = simple sequence repeats (SSR)] have been recognized as one of the most popular and ideal technologies for unfolding genetic variation among fungal pathogens because of their ubiquitous nature, high polymorphism, co-dominance inheritance, and high level of allelic variation within the genome (Kumar et al., 2012; Singh et al., 2014; Kashyap et al., 2015; Rai et al., 2016; Savadi et al., 2020). Several research studies indicated the potential of microsatellites in dissecting the population genetic structure and defining the evolutionary relationships among myriads of fungi responsible for causing smut and bunt diseases in plants (Zhou et al., 2008; Zhang et al., 2015; Sharma et al., 2018; Kashyap et al., 2019b). Currently, few reports exist regarding the application of microsatellites in exploring genetic variation in *T. indica* (Kaur et al., 2015; Sharma et al., 2018; Gurjar et al., 2022). Moreover, no database has been developed that can provide information related to the distribution and dynamics of microsatellite markers in the *T. indica* genome. In recent time, the genomes of nine isolates of *T. indica* (PSWKBGH_1, PSWKBGH_2, PSWKBGD_1_3, RAKB_UP_1, TiK_1, Tik, DAOMC236408, DAOMC236414, and DAOMC236416) have been decoded, and information about them is available in the public domain.^1^ Keeping the aforementioned points in mind, current research has been initiated to mine the multiple genomic resources of *T. indica* for the discovery and characterization of microsatellite-based markers. The prime objectives of the study include (i) the investigation of nine different genomes of *T. indica* for finding out the distribution pattern and dynamics of microsatellites at inter-and intra-genome levels, (ii) the identification and validation of microsatellite-derived markers for dissecting genetic variation in *T. indica* isolates, and (iii) the assessment of diversity and structure of the *T. indica* population by polymorphic microsatellite markers.

## Materials and methods

### *Tilletia indica* isolates and culture conditions

The study was made on a set of fifty isolates of *T. indica* representing different geographical regions of North India (Table 1). *T. indica* isolates were isolated from KB-infected grain samples collected during 2019–2020 from grain mandies in seven different regions of North India (Haryana, Rajasthan, Punjab, Uttar Pradesh, Uttarakhand, Jammu, and Himachal Pradesh). Teliospores of each isolate were extracted by puncturing a sorus of *T. indica-*infected seed, and spores were processed for germination at 121°C in a Petri-plate amended with 2% water agar (HiMedia, India). It is important to mention that a single germinating teliospore was chosen in random fashion from a Petri-plate containing water agar with the help of a sterilized needle. The selected spore was further placed on a Petri-plate amended with potato dextrose agar (PDA; HiMedia, India) and incubated at 18 ± 2°C for 2 weeks under alternate cycles of dark and light conditions before executing further experiments.

**Table 1:** Description of *Tilletia indica* isolates collected from different states of North India.

Isolate(s)	Location	Year of collection	NCBI gene bank accession No.	Coefficient of infection (%) after artificial inoculations		
				WL711	WH 542	PBW343
KTi-19-1	Punjab	2019	MT497985	39.62 ± 3.65^i^	36.40 ± 3.95^ij^	42.23 ± 1.05^o^
KTi-19-2	Punjab	2019	MT497986	17.04 ± 1.57 ^d^	17.65 ± 2.12^de^	14.23 ± 0.81 ^f^
KTi-19-3	Punjab	2019	MT497987	18.60 ± 2.09 ^d^	18.47 ± 3.71^e f^	17.48 ± 0.69^ij^
KTi-19-4	Punjab	2019	MT497988	15.02 ± 3.25^cd^	15.37 ± 3.54^d^	17.64 ± 0.53^ij^
KTi-19-5	Punjab	2019	MT497989	20.70 ± 3.74^de^	21.33 ± 2.95^ef^	26.10 ± 0.52^lm^
KTi-19-6	Rajasthan	2019	MT497990	17.36 ± 4.71 ^d^	18.11 ± 2.25^e^	16.10 ± 0.43 ^g^
KTi-19-7	Uttarakhand	2019	MT497991	16.90 ± 1.62 ^d^	17.22 ± 2.35 ^d^	18.83 ± 0.77^j^
KTi-19-8	Uttar Pradesh	2019	MT497992	18.55 ± 1.25 ^d^	18.67 ± 3.33 ^f^	17.23 ± 0.29^ij^
KTi-19-9	Himachal Pradesh	2019	MT497993	16.22 ± 2.34^d^	16.12 ± 2.14^d^	18.28 ± 0.38^jk^
KTi-19-10	Rajasthan	2019	MT497994	26.99 ± 2.33^f^	28.90 ± 3.65^gh^	24.01 ± 0.86^l^
KTi-19-11	Rajasthan	2019	MT497995	15.80 ± 1.25^cd^	17.74 ± 1.61^d^	15.82 ± 0.79^g^
KTi-19-12	Jammu	2019	MT497996	29.78 ± 4.34^de^	22.24 ± 2.72^f^	27.47 ± 0.85^lmn^
KTi-19-13	Punjab	2019	MT497997	15.96 ± 2.22^cd^	17.24 ± 1.12 ^d^	17.19 ± 0.43^i^
KTi-19-14	Rajasthan	2019	MT497998	13.93 ± 1.32 ^c^	11.64 ± 3.11^b^	16.92 ± 0.22 ^h^
KTi-19-15	Haryana	2019	MT497999	13.71 ± 1.14 ^c^	11.46 ± 2.32^b^	16.65 ± 0.24 ^g^
KTi-19-16	Jammu	2019	MT498000	13.50 ± 1.15 ^c^	11.28 ± 1.41^b^	16.38 ± 0.59^gh^
KTi-19-17	Punjab	2019	MT498001	43.28 ± 1.22^j^	41.10 ± 4.33^jk^	46.11 ± 1.02^p^
KTi-19-18	Haryana	2019	MT498002	35.64 ± 1.04^h^	38.65 ± 3.13^ijk^	36.83 ± 1.01^n^
KTi-19-19	Uttarakhand	2019	MT498003	14.42 ± 1.07^c^	17.48 ± 1.27 ^d^	15.56 ± 1.14 ^g^
KTi-19-20	Jammu	2019	MT498004	15.43 ± 1.04 ^c^	19.00 ± 1.29 ^e f^	17.29 ± 1.18^ij^
KTi-19-21	Punjab	2019	MT498005	12.40 ± 3.09^c^	10.37 ± 2.28^ab^	15.02 ± 1.17^fg^
KTi-19-22	Punjab	2019	MT498006	18.18 ± 1.75 ^d^	17.19 ± 2.31^d^	19.75 ± 2.15^jk^
KTi-19-23	Uttarakhand	2019	MT498007	31.96 ± 1.44^g^	30.01 ± 4.26^hi^	38.47 ± 3.13^mn^
KTi-19-24	Haryana	2019	MT498008	23.32 ± 2.37^e^	24.56 ± 3.29^g^	24.20 ± 2.72 ^L^
KTi-19-25	Punjab	2019	MT498009	23.07 ± 2.36^e^	24.29 ± 2.34^g^	33.93 ± 3.66^n^
KTi-19-26	Punjab	2019	MT498010	12.83 ± 1.43^c^	14.03 ± 1.44^c^	13.66 ± 1.63^f^
KTi-19-27	Punjab	2019	MT498011	13.14 ± 2.31 ^c^	14.17 ± 3.81^c^	13.39 ± 1.61^e^
KTi-19-28	Uttar Pradesh	2019	MT498012	24.65 ± 1.69^e^	15.22 ± 2.62^d^	13.11 ± 1.48^e^
KTi-19-29	Haryana	2019	MT498013	8.94 ± 1.70 ^a^	7.64 ± 1.32^a^	12.84 ± 1.49^e^
KTi-19-30	Uttar Pradesh	2019	MT498014	7.40 ± 1.94^a^	7.17 ± 0.92 ^a^	6.23 ± 0.92^a^
KTi-19–31	Uttar Pradesh	2019	MT498015	15.21 ± 1.35^c^	14.56 ± 1.52^cd^	12.30 ± 1.31^e^
KTi-19-32	Rajasthan	2019	MT498016	19.55 ± 2.75^de^	21.05 ± 2.14^ef^	19.03 ± 2.02^jk^
KTi-19-33	Haryana	2019	MT498017	9.31 ± 0.92^a^	8.20 ± 0.16^ab^	6.57 ± 0.22^a^
KTi-19-34	Punjab	2019	MT498018	9.15 ± 0.22^a^	8.02 ± 0.37^a^	7.48 ± 0.71^ab^
KTi-19-35	Rajasthan	2019	MT498019	10.33 ± 0.52^b^	11.84 ± 0.09^b^	14.21 ± 0.68^f^
KTi-19-36	Punjab	2019	MT498020	9.11 ± 0.75^a^	7.65 ± 0.62 ^a^	9.24 ± 0.32^d^
KTi-19-37	Rajasthan	2019	MT498021	8.89 ± 0.43^a^	7.47 ± 0.92 ^a^	9.67 ± 0.24^d^
KTi-19-38	Himachal Pradesh	2019	MT498022	8.68 ± 0.31 ^a^	8.47 ± 0.26^ab^	8.39 ± 0.45 ^b^
KTi-19-39	Himachal Pradesh	2019	MT498023	8.46 ± 0.29 ^a^	8.61 ± 0.12^ab^	9.12 ± 0.72^cd^
KTi-19-40	Rajasthan	2019	MT498024	8.24 ± 0.24 ^a^	7.93 ± 0.65 ^a^	9.85 ± 0.05^d^
KTi-19-41	Rajasthan	2019	MT498025	7.87 ± 0.44 ^a^	7.75 ± 1.92 ^a^	8.98 ± 0.52^c^
KTi-19-42	Uttar Pradesh	2019	MT498026	16.49 ± 1.21^d^	13.23 ± 1.42^c^	18.51 ± 0.12^jk^
KTi-19-43	Uttar Pradesh	2019	MT498027	8.38 ± 0.22 ^a^	8.74 ± 0.25^ab^	9.03 ± 0.42^cd^
KTi-19-44	Jammu	2019	MT498028	7.85 ± 0.15 ^a^	8.67 ± 0.26^ab^	8.76 ± 0.26 ^b^
KTi-19-45	Jammu	2019	MT498029	10.70 ± 0.65^b^	18.69 ± 0.52^e f^	16.49 ± 0.29 ^g^
KTi-19-46	Jammu	2019	MT498030	8.70 ± 0.45 ^a^	7.29 ± 1.61^a^	8.22 ± 0.32 ^b^
KTi-19-47	Himachal Pradesh	2019	MT498031	9.17 ± 0.39 ^a^	8.76 ± 0.10^ab^	7.95 ± 0.94^b^
KTi-19-48	Himachal Pradesh	2019	MT498032	23.15 ± 2.99^ef^	30.48 ± 3.97^hi^	27.67 ± 3.12^mn^
KTi-19-49	Haryana	2019	MT498033	24.70 ± 2.56^ef^	20.96 ± 2.42^f^	25.97 ± 4.62^klm^
KTi-19-50	Himachal Pradesh	2019	MT498034	13.26 ± 2.63^c^	12.12 ± 1.32^b^	15.67 ± 1.52^fg^

Coefficient of infection (CI) = [(0·25 × seeds in grade 0·1 to 1) + (0·50 × seeds in grade 2) + (0·75 × seeds in grade 3) + (1·0 × seeds in grade 4)] × 100/total number of grains HA: Highly aggressive (CI = > 20.0%); MA = Moderately aggressive (CI = 10.0–20.0%); LA: Least aggressive (CI = <10.0%).

### Aggressiveness and virulence assessment

The aggressive nature of *T. indica* isolates was studied by inoculating each isolate independently on three susceptible wheat cultivars (WL711, WH542, and PBW343). The seeds were grown in one-meter-long strips with a strip-to-strip distance of 25 cm during the *rabi* cropping season (2021–2022) at the experimental field of the ICAR-Indian Institute of Wheat and Barley Research (IIWBR), Karnal, India. Three replicates of each genotype were maintained. The wheat sowing operation was performed during the second week of November and was similar to the period of normal sowing of wheat in North India. Bulk inocula of each *T. indica* isolate producing secondary sporidia (allantoids) were raised on PDA containing Petri-plates. The load of the liquid suspension of secondary sporidia (6 × 10^6^ mL^−1^) was optimized with a hemocytometer. During evening hours, two milliliter of standardized liquid suspension of each isolate in the ear-head was inserted with the help of a hypodermal syringe (Aujla et al., 1989) in ten main tillers of each cultivar (i.e., WL711, WH 542 and PBW343) at Zadock’s growth stage (ZGS 49, i.e., boot leaf stage) (Zadocks et al., 1974) (Supplementary Figure S1). A single sterilized syringe per isolate was employed to avoid the cross-contamination of KB isolates among each other. After inoculation, each inoculated tiller was tagged. An environment of high humidity (>70%) was regularly maintained by performing mist sprays at regular intervals of 4 h. At crop maturity, inoculated ear heads were handpicked and threshed. Every seed of the inoculated tiller was visually examined. In the case of point infections in the seeds, a magnifying lens or a microscope was used to confirm the presence of KB teliospores. Data pertaining to the number of KB-infected grains per inoculated ear as well as their level of infection per grain was also recorded. The numerical values of 0, 0.25, 0.50, 0.75, and 1.0 were used to indicate the infection severity (infection grade) of 0, 1, 2, 3, and 4, respectively. The percent coefficient of infection (CI) was computed by employing the below-mentioned formula.


$$\mathrm{CI}=\sum \frac{X_{i}Y_{i}}{N}\times 100$$

Where, CI = Per cent coefficient of infection; N = Numbers of total grains analyzed; i = infection severity grade (i = 0 to 4); X = Numerical value of ith grade of infection severity; and Y = Total number of grains of i^th^ grade of infection severity.

The obtained CI values were further used to categorize aggressivity of each isolate inoculated on susceptible cultivars (WL711, WH542 and PBW343). All the *T. indica* isolates under study were further classified into three major groups. These includes: highly aggressive (HA) isolates (CI >20%), moderately aggressive (MA) isolates (CI ranged between 10–20%) and least or weakly aggressive (LA) isolates (CI <10%).

### *Tilletia indica* genomic resources and computational analysis

The sequence data used in the current study was collected from nine different whole genome sequences (PSWKBGH_1, PSWKBGH_2, PSWKBGD_1_3, RAKB_UP_1, TiK_1, Tik, DAOMC236408, DAOMC236414, and DAOMC236416) available in the public domain (NCBI; https://www.ncbi.nlm.nih.gov/genome/browse/#!/eukaryotes/8345/) for the exploration of microsatellite rpeat motifs. The retrieved data was assessed on different parameters such as motif occurrence frequency, relative density (RD) of repeat motifs, and relative abundance (RA) of repeat motifs with the help of Krait software (Du et al., 2018). The numerical value setting criteria used to discover different microsatellite loci were fixed at 12 for mono-repeat motifs, followed by 7 for di-repeat motifs, 5 for tri-repeat motifs, and 4 for the remaining tetra-, penta-, and hexa-repeat motifs. A random selection of fifty SSR primers from all nine genomes of *T. indica* was performed before amplified product validation using a polymerase chain reaction (PCR) assay. PRIMER3 online software^2^ was used to develop and select primers for PCR assays.

### PCR amplification and SSR genotyping

The genomic DNA of all 50 isolates of *T. indica* was isolated using the cetyl trimethylammonium bromide (CTAB)-based protocol of Kumar et al. (2013). The quality and quantity of extracted genomic DNA from each isolate were determined using Scandrop^2^ spectrophotometers (Analytik Jena, Germany). A PCR assay was conducted in a total of 25 μL of reaction and executed in a Q Cycler 96 (Hain Lifescience, United Kingdom) machine for amplification of each SSR locus marker. The PCR master reaction was prepared by incorporating *T. indica* DNA (50 ng μ1), GoTaq green master mix (12.5 μL; Promega, United States), and 1 μL of each primer (10 M) in a thin-walled PCR tube (Genaxy, India). The final reaction volume (25 μL) was fixed with the help of sterilized distilled water. The thermocycling program runs after setting the preliminary denaturation temperature at 95°C for 2 min, followed by six touch-down PCR cycles comprising 95°C for 20 s, 57/53°C for 15 s, and 72°C for 30 s. These cycles were followed by 40 cycles of denaturation at 95°C for 20 s with an invariable annealing temperature of 57 or 53°C (depending on the marker as mentioned in Table 2) for 15 s, extension at 72°C for 30 s, and a final elongation step at 72°C for 30 min. All the amplified products were visualized on a 3.5% agarose gel using ethidium bromide staining. A DNA ladder (100 bp; Promega, USA) was employed to compare and estimate the size of the amplified product.

**Table 2:** Details of primer sequences, motifs, annealing temperatures (T_a_), and other indices of polymorphic simple sequence repeat (SSR) markers in the 50 geographical distinct *Tilletia indica* isolates.

Marker	Sequence (5′-3′)	Motif	T_a_ (°C)	Alleles (AS)	He	PIC
TiSSR10	F:CTGTAGATGATGGGCCCATTCC	(CCT)_5_	54	2 (170–180)	0.50	0.37
	R:GATTATCTATATGCGGTCACGGC					
TiSSR17	F:TGTACTGCTGACATCTCTCTCC	(CTT)_7_	56	3 (130–280)	0.62	0.55
	R:GTATGGTGCTTTGTCGAGTTCG					
TiSSR19	F:TGTAGTACCAGCATCCAAGAGC	(CCT)_3_	53	2 (150–170)	0.50	0.37
	R:GAAAATGGCGAATCGGATGAGG					
TiSSR20	F:GCCGTTCGAAGTTGATATCTTGC	(TCG)_5_	53	2 (120–140)	0.50	0.37
	R:ACAGCCTTCTTCATCTTCCAGG					
TiSSR27	F:TCTGGCTATTACCACTGTTCACC	(TAGTCA)_3_	54	7 (180–580)	0.83	0.81
	R:CAGTGATCGGCGTGACTATGG					
TiSSR40	F:GACATCATCGCCCAACAAATCG	(GTC)_2_	54	2 (170–210)	0.50	0.37
	R:TCTCAATCCCCTCTTTTCTCGC					
TiSSR41	F:CCCATCCACATTCACACAAACC	(ACCC)_3_	54	2 (165–185)	0.50	0.37
	R:TGGTGGCGAAATAGACTCACC					
TiSSR42	F:AGCGGAAGAATGAGAGCATAGG	(AGG)_4_	53	2 (155–175)	0.50	0.37
	R:CGGAAGGAGGTAGTAAGGAAGG					
TiSSR45	F:ATACCATGTGAAAGAGAGGCCG	(AGA)_2_	52	2 (165–195)	0.50	0.37
	R:ATAGAACCGGTTTTCTCCTCGG					
TiSSR47	F:TCCCGACTATCATACAACCACC	(CCT)_10_	52	2 (110–140)	0.50	0.37
	R:CTTCGTTGACTGTGAGGTCTCC					

He, Expected heterozygosity; PIC, Polymorphism information content; AS, amplicon size in base pair.

### Statistical analysis

Each *T. indica* isolate was monitored for the presence (recorded as 1) or absence (recorded as 0) of amplified products by each SSR primer used in the PCR assay. The 0/1 matrix was used to compute the similarity genetic distance using the Simqual option available in the computer-driven numerical taxonomy and multivariate analysis system (NTSYS) software, version 2.1 (Rohlf, 2002). To deduce the genetic relationships among different isolates of *T. indica*, the resultant similarity coefficients were taken into consideration for the generation of a dendrogram based on the unweighted paired group method of arithmetic averages (UPGMA) algorithm and sequential agglomerative hierarchical non-overlapping (SAHN) grouping. The computation of heterozygosity (He) and polymorphism information content (PIC) was made according to Botstein et al. (1980). The PIC value was determined by using the below-mentioned formula:


$${\mathrm{PIC}}_{i}=1-\overset{n}{\underset{j=1}{\sum }}{\mathrm{Pij}}^{2}$$

where Pij depicts frequency of the jth allele for the marker i allessles.

Analysis of molecular variance (AMOVA) was computed by using GenAlEx 6.5 (Peakall and Smouse, 2012) to figure out the role of variance components in genetic variation at the inter-and intra-population levels. Population structure was determined by Structure 2.3.4 (Pritchard et al., 2000). The STRUCTURE program was run by giving command of five independent runs of 50,000 burns in period length at fixed iterations of 1,00,000. Further, the methodology of Evanno et al. (2005) was referred to fix the optimum K-value. Besides this, field experiments performed to check the aggressiveness of each *T. indica* isolate were statistically arranged in a randomized block design (RBD) with three independent replicates. An analysis of variance (ANOVA) was conducted to test the significance of the generated data. Duncan’s multiple range test (DMRT) is used to make *post hoc* comparative analyzes of the mean data.

## Results

### Aggressiveness assessment of *Tilletia indica* isolates

**Figure 1:**
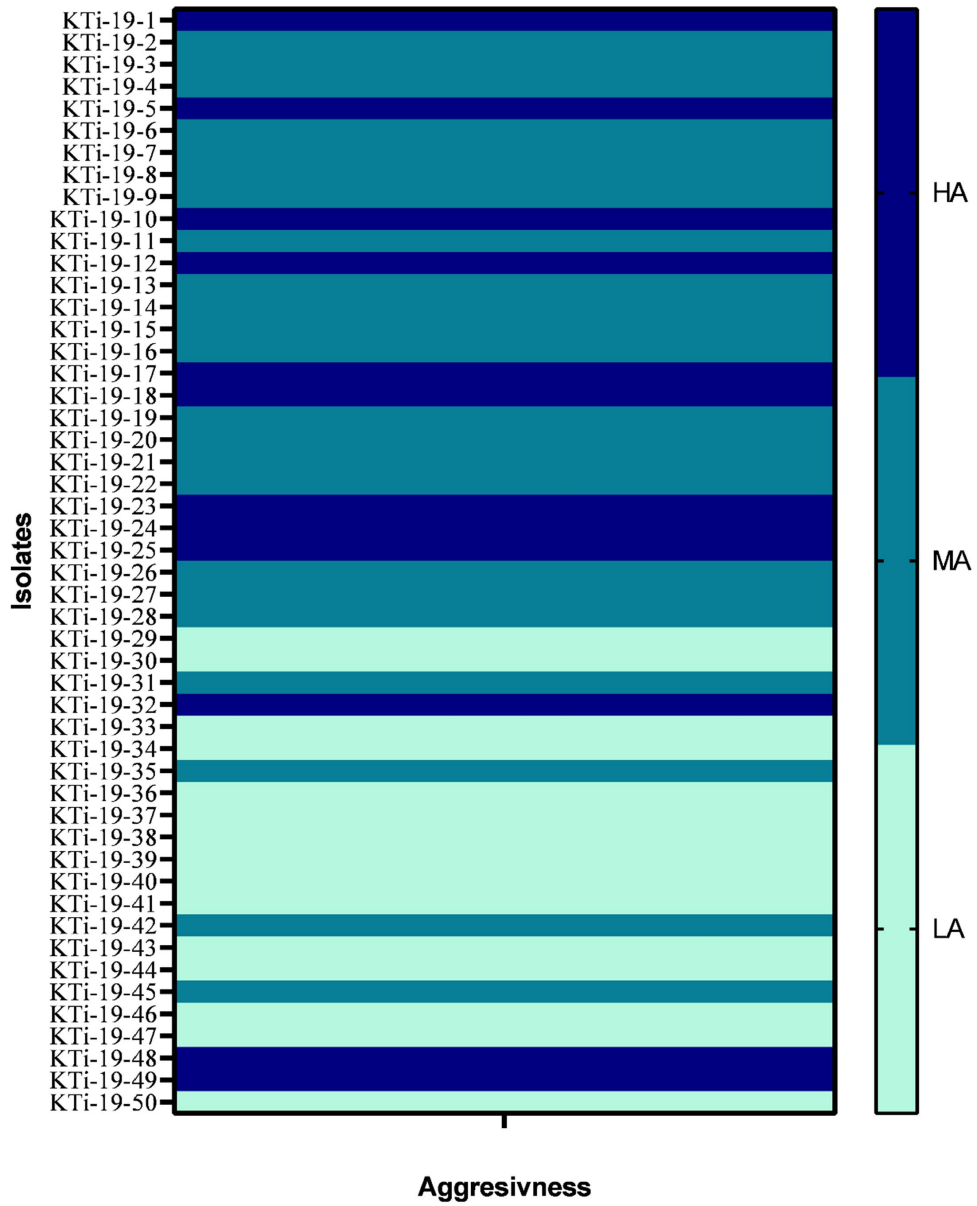
Heat map showing aggressiveness of *Tilletia indica* isolates. HA: Highly aggressive (CI = 20%), MA: moderately aggressive (CI = 10–20%), LA (least or weakly aggressive = CI <10%).

All the 50 isolates of *T. indica* were assessed on the parameter of their aggressivity on three susceptible wheat cultivars (cv. WL711, WH542 and PBW343) and obtained data was presented in Table 1. The range of CI in all the three cultivars *viz*., WL711, WH542 and PBW343 was 7.40–43.28%, 7.17–41.10% and 6.23–46.11%, respectively (Table 1). Isolate KTi-19-17 was found highly aggressive in nature as revealed by CI values more than 41% in all the three cultivars. Similarly, KTi-19-30 was found least aggressive as lowest CI was recorded in WL711 (7.40%) followed by WH542 (7.17%) and PBW343 (6.23%) cultivars. Further, it was noticed that the aggressivity of tested KB isolates ranged from HA (30% *T. indica* isolates) to LA (24% *T. indica* isolates) and MA (46% *T. indica* isolates) (Figure 1).

### Genome-wide distribution patterns of microsatellite repeats

Nine distinct *T. indica* whole genome sequences were mined to determine the total lengths of all kinds of motifs per megabase pair (Mbp) of DNA sequence in order to evaluate the importance of motif length to microsatellite prevalence (Table 3). The PSWKBGD_1_3 genome was found to have the most microsatellites (7336), followed by the PSWKBGH_1 and PSWKBGH_2 genomes (6,426 and 6,328, respectively), DAOMC236408 (5022), RAKB_UP_1 (4915), TiK_1 (4880), DAOMC236416 (4756), DAOMC236414 (4437), and Tik (4224). DAOMC236414 (98.38%) had the highest proportion of perfect microsatellites, followed by DAOMC236416 (98.04%), DAOMC236408 (98.01%), Tik (97.49%), PSWKBGH_1 (97.42%), TiK_1 (97.34%), RAKB_UP_1 (97.21%), PSWKBGH_2 (96.84%), and PSWKBGD_1_3 (95.24%). In addition, it was discovered that the PSWKBGH_1 (171.54) genome had the highest relative abundance of microsatellites when compared to the PSWKBGH_2 (170.03), DAOMC236408 (169.29), PSWKBGD_1_3 (167.92), DAOMC236416 (164.35), Tik (158.17), DAOMC236414 (153.19), TiK_1 (153.311), and RAKB_UP_1 (145). Similar to this, RD of SSR was seen to be at its highest in PSWKBGD_1_3 (3938.8), followed by PSWKBGH_2 (3457.92), PSWKBGH_1 (3397.81), DAOMC236408 (3275.61), DAOMC236416 (3251.37), Tik (3150.31), TiK_1 (2993.85), and RAKB_UP_1 (2885.01) and DAOMC236414 (2840.49). (Table 3). Table 4 contains detailed information on the percentage, relative abundance (RA), and relative density (RD) of SSRs in sequence sets from various *T. indica* isolates.

**Table 3:** Number and distribution of SSRs in different isolates of *Tilletia indica.*

Isolate	PSWKBGH_1	PSWKBGH_2	PSWKBGD_1_3	RAKB_UP_1	TiK_1	Tik	DAOMC236408	DAOM236414	DAOM236416
Origin	India	India	India	India	India	India	Canada	Canada	Canada
GS (Mb)	37.5	37.2	43.7	33.8	31.8	26.7	29.7	29	29
% G + C	54.63	54.68	54.67	55.24	54.79	53.99	54.84	55.02	54.92
TSSR	6,426	6,328	7,336	4,915	4,880	4,224	5,022	4,437	4,756
pSSR	6,260 (97.42%)	6,128 (96.84%)	6,987 (95.24%)	4,778 (97.21%)	4,750 (97.34%)	4,118 (97.49%)	4,922 (98.01%)	4,365 (98.38%)	4,663 (98.04%)
cSSR	166 (2.58%)	200 (3.16%)	349 (4.76%)	137 (2.79%)	130 (2.66%)	106 (2.51%)	100 (1.99%)	72 (1.62%)	93 (1.96%)
TL	127,283	128,693	172,077	97,430	95,298	84,132	97,173	82,270	94,086
RA	171.54	170.03	167.92	145.54	153.311	158.17	169.29	153.19	164.35
RD	3397.81	3457.92	3938.8	2885.01	2993.85	3150.31	3275.61	2840.49	3251.37

GS, Genome Size; RA, Relative abundance; RD, Relative density; TL, Total length of SSR; TSSR, Total number of SSR; pSSR, Perfect SSR; cSSR, Compound SSR.

**Table 4:** Percentage, relative abundance, and relative density of SSRs in sequence sets of different isolates of *Tilletia indica.*

Isolate(s)	Motif type	Counts	AL (bp)	RA (loci/Mb)	RD (bp/Mb)
PSWKBGH_1	Mono	1,386	17.94	37	663.66
	Di	947	18.58	25.28	469.67
	Tri	2,788	18	74.43	1339.34
	Tetra	654	21.16	17.46	369.46
	Penta	164	24.82	4.38	108.65
	Hexa	487	34.39	13	447.03
PSWKBGH_2	Mono	1,433	17.79	38.5	685.07
	Di	973	18.32	26.14	478.98
	Tri	2,630	18.09	70.67	1278.29
	Tetra	608	23.96	16.34	391.44
	Penta	166	26.11	4.46	116.48
	Hexa	518	36.47	13.92	507.67
PSWKBGD_1_3	Mono	1917	35.94	43.88	1577.22
	Di	1,101	17.37	25.2	437.79
	Tri	3,063	17.51	70.11	1227.46
	Tetra	641	20.94	14.67	307.27
	Penta	193	24.43	4.42	107.93
	Hexa	421	29.17	9.64	281.13
RAKB_UP_1	Mono	610	21.75	18.06	392.82
	Di	867	18.02	25.67	462.58
	Tri	2,456	17.5	72.72	1272.62
	Tetra	480	20.04	14.21	284.86
	Penta	141	25.89	4.18	108.08
	Hexa	361	34.06	10.69	364.04
TiK_1	Mono	618	20.83	19.41	404.41
	Di	859	18.03	26.99	486.57
	Tri	2,449	17.47	76.94	1343.96
	Tetra	474	19.84	14.89	295.43
	Penta	138	26.99	4.34	117.02
	Hexa	342	32.25	10.74	346.45
Tik	Mono	942	18.15	35.27	640.38
	Di	610	17.92	22.84	409.27
	Tri	1894	17.88	70.92	1268.37
	Tetra	346	20.21	12.96	261.81
	Penta	114	24.43	4.27	104.28
	Hexa	318	39.15	11.91	466.19
DAOMC236408	Mono	953	18.97	32.12	609.56
	Di	805	18.5	27.14	501.93
	Tri	2,399	17.58	80.87	1421.55
	Tetra	465	20.83	15.67	326.44
	Penta	106	27.59	3.57	98.6
	Hexa	294	32.04	9.91	317.54	DAOMC236414	Mono	438	18.03	15.12	272.72		Di	790	17.56	27.28	478.95	
Tri	2,406	17.36	83.07	1442.13	
Tetra	437	19.66	15.09	296.65	
Penta	91	23.52	3.14	73.89	
Hexa	275	29.08	9.49	276.14
DAOMC 236416	Mono	800	22.25	27.65	615.02
	Di	768	18.52	26.54	491.61
	Tri	2,344	17.55	81	1421.24
	Tetra	464	20.07	16.03	321.8
	Penta	100	27.8	3.46	96.07
	Hexa	280	31.59	9.68	305.63

**Figure 2:**
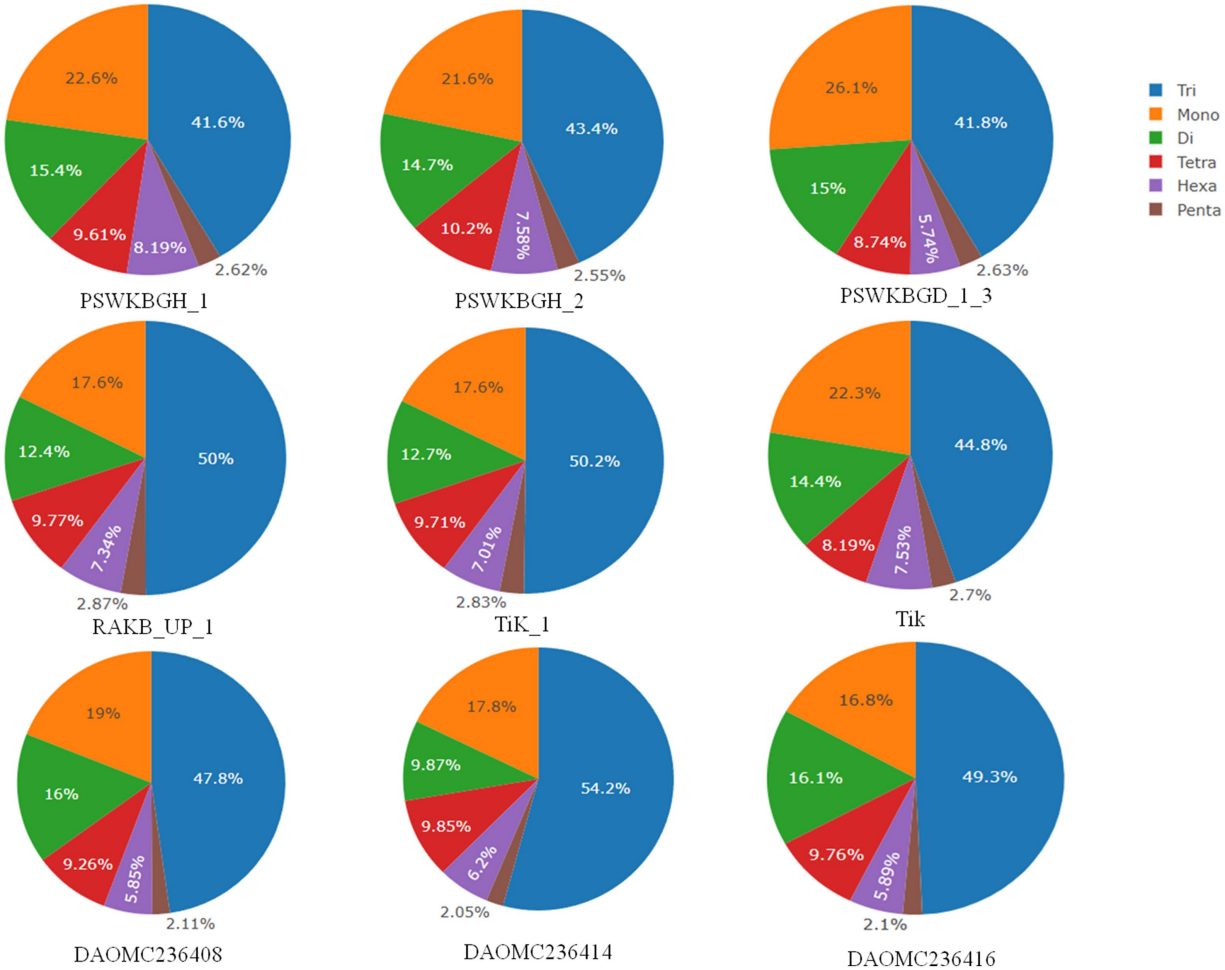
SSR count distribution for each type in *Tilletia indica* genomes.

**Figure 3:**
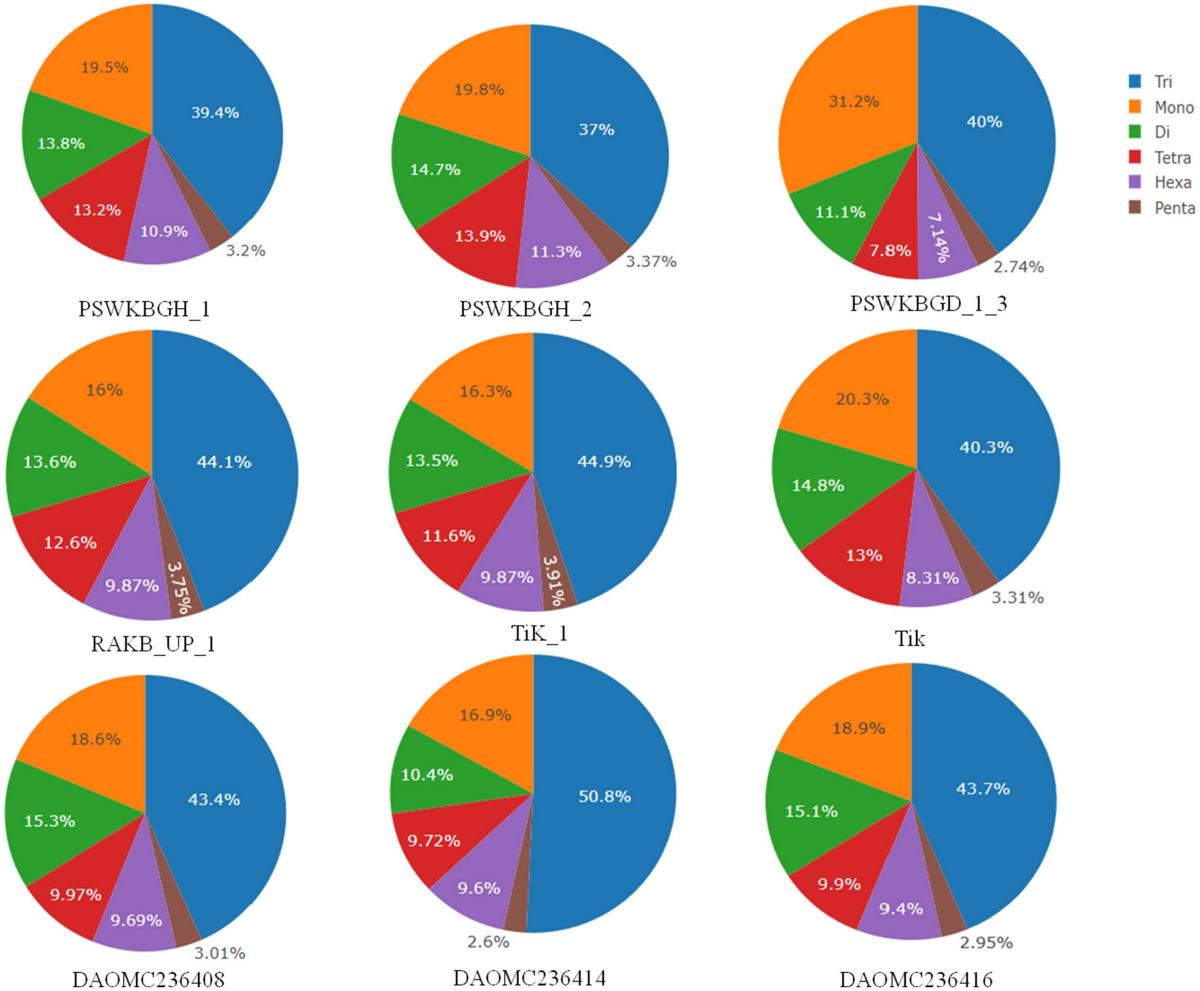
SSR length distribution for each motif in *Tilletia indica* genomes.

**Figure 4:**
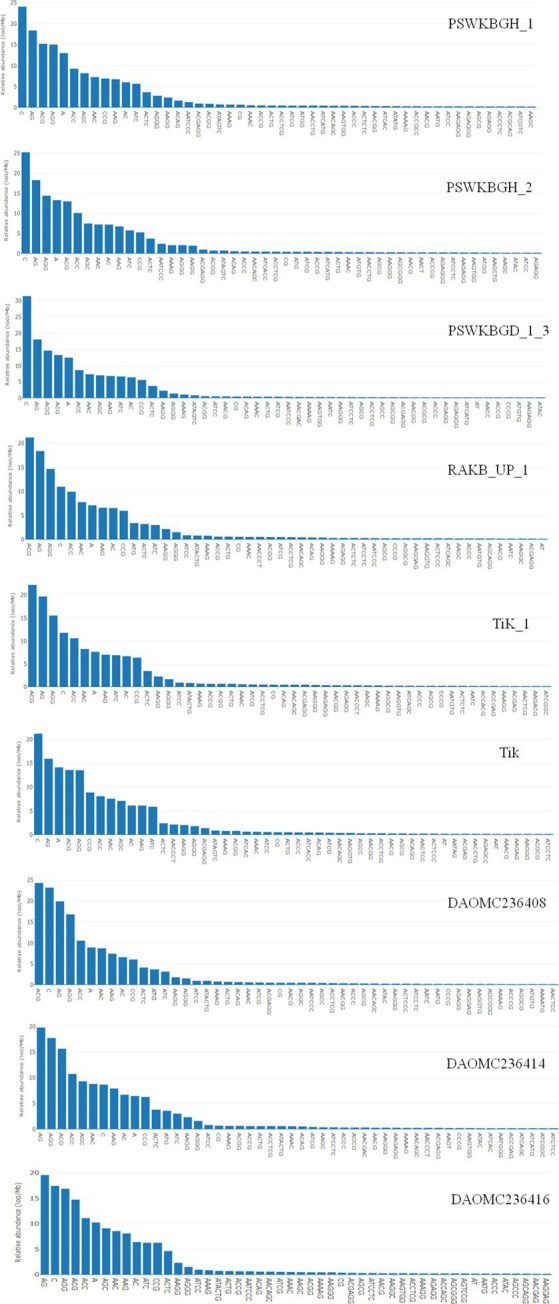
Distribution of most abundant motifs in *Tilletia indica* genomes.

A total of 2344 (49.3% of the genome), 2406 (54.2% of the genome), 2399 (47.8% of the genome), 2788 (41.6% of the genome), 3063 (41.8% of the genome), 2630 (43.4% of the genome), 2456 (50% of the genome), 2449 (50.2% of the genome), and 1894 (44.8% of the genome) tri-nucleotide motif types were identified in DAOMC236416, DAOMC236414, DAOMC236408, PSWKBGH_1, PSWKBGD_1_3_3, PSWKBGH_2, RAKB_UP_1, TiK_1 and Tik, respectively (Table 4). On the basis of microsatellite count distribution, tri-nucleotide repeat units followed by mono-, di-, tetra-, hexa-, and penta-nucleotide repeat motifs were predominant in DAOMC236416, DAOMC236408, PSWKBGD_1_3, PSWKBGH_2, and Tik genomes (Table 4; Figure 2). Contrarily, DAOMC236414, PSWKBGH_1, RAKB_UP_1, and TiK_1 genomes showed the dominance of tri-nucleotide motifs, followed by mono-, di-, tetra-, hexa-, and penta-nucleotide motifs. A similar trend was observed in all the genomes when the SSR length distribution for each type of motif was explored in all the genomes (Figure 3). The most frequent motif in DAOMC236414, DAOMC236408, PSWKBGH_1, RAKB_UP_1, TiK_1, and Tik genomes was ACG, except in genome DAOMC236416, PSWKBGD_1_3 and PSWKBGH_2, where AGG was found to be the most frequent repeat. Overall, the repeats of AG, AGG, ACG, ACTC, AAAAG, AAGGG, AACGG, ATGTG, ATCAC, ATACTG, ACCTCG, ATAGTC, AATCCC, and AACCCT were abundant in all the genomes (Table 5). The C/G motif in all the genomes was the most abundant mono-nucleotide motif (Figure 4).

**Table 5:** The longest SSR motif found in the transcript sequences of *Tilletia indica* isolates.

Isolate	Nucleotide repeats (Motifs)				
	Di	Tri	Tetra	Penta	Hexa
PSWKBGH_1	(AG)_687 (10.77%)_	(ACG)_569 (8.85%)_	(ACTC)_137 (2.13%)_	(AACGG)_14 (0.22%)_	(AATCCC)_49 (0.76%)_
PSWKBGH_2	(AG)_680 (10.75%)_	(AGG)_536 (8.47%)_	(ACTC)_139 (2.2%)_	(ATGTG)_14 (0.22%)_	(ATAGTC)_29 (0.46%)_
PSWKBGD_1_3	(AG)_789 (10.76%)_	(AGG)_640 (8.72%)_	(ACTC)_164 (2.24%)_	(AAGGG)_15 (0.2%)_	(ATAGTC)_39 (0.53%)_
RAKB_UP_1	(AG)_620 (12.61%)_	(ACG)_715 (14.55%)_	(ACTC)_109 (2.2%)_	(AAGGG)_14 (0.28%)_	(ATACTG)_28 (0.57%)_
TiK_1	(AG)_626 (12.83%)_	(ACG)_706 (14.47%)_	(ACTC)_110 (2.25%)_	(AAGGG)_14 (0.29%)_	(ATACTG)_28 (0.57%)_
Tik	(AG)_425 (10.06%)_	(ACG)_362 (8.57%)_	(ACTC)_65 (1.54%)_	(ATCAC)_18 (0.43%)_	(AACCCT)_58 (1.37)_
DAOMC236408	(AG)_591 (11.77%)_	(ACG)_721 (14.36%)_	(ACTC)_123 (2.45%)_	(AACGG)_12 (0.24%)_	(ATACTG)_29 (0.58%)_
DAOMC236414	(AG)_572 (12.89%)_	(ACG)_513 (11.56%)_	(ACTC)_110 (2.48%)_	(AAGGG)_9 (0.2%)_	(ACCTCG)_18 (0.41%)_
DAOMC236416	(AG)_566 (11.9%)_	(AGG)_488 (10.26%)_	(ACTC)_134 (2.82%)_	(AAAAG)_13 (0.27%)_	(ATACTG)_21 (0.44%)_

### Development of genome-wide microsatellite markers and polymorphism evaluation

Among fifty microsatellite markers, only 36 SSR markers were able to generate amplicons when tested on the genomic DNA of *T. indica*. However, only ten loci showed polymorphism among all 50 isolates and displayed well-amplified and easily detectable amplicons ranging from 110 to 580 bp (Table 2). Among amplified markers, ten markers (37.5%) were polymorphic (PIC >0.35%), and the remaining 26 markers showed monomorphic alleles. A total of 26 alleles were amplified by ten markers (Table 2). Maximum alleles (7) were amplified by the TiSSR27 marker. Both TiSSR17 and TiSSR27 found the most informative SSR markers based on their PIC values (>0.50) and heterozygosity values (>0.62) (Table 2).

### Diversity and cluster analysis

**Figure 5:**
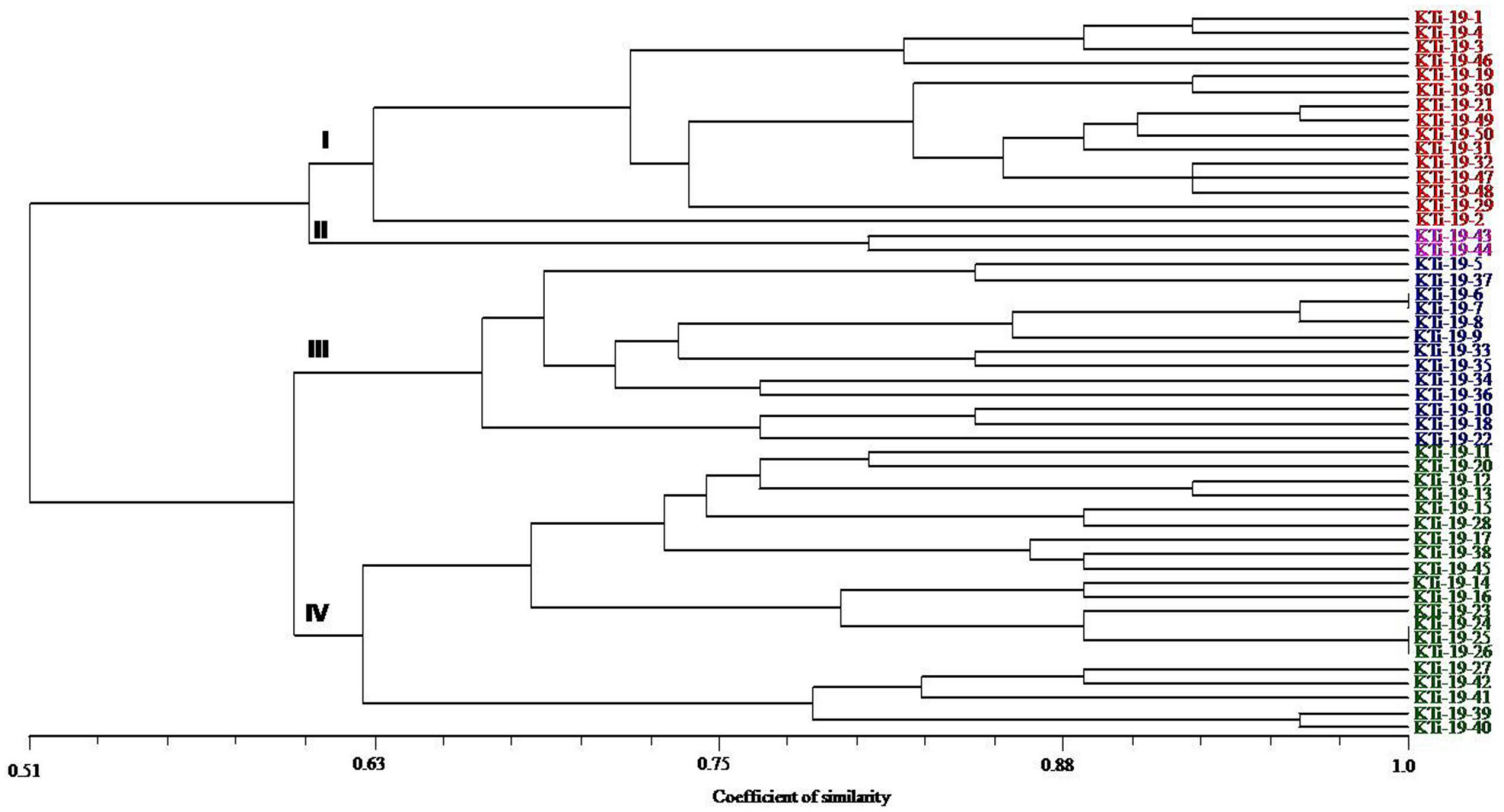
Dendrogram generated by adopting UPGMA clustering method among 50 isolates of *Tilletia indica* using 10 polymorphic microsatellite markers. The scale in the figure is genetic similarity coefficient computed according to Jaccard’s. Numbers at the nodes represent cluster groups.

**Figure 6:**
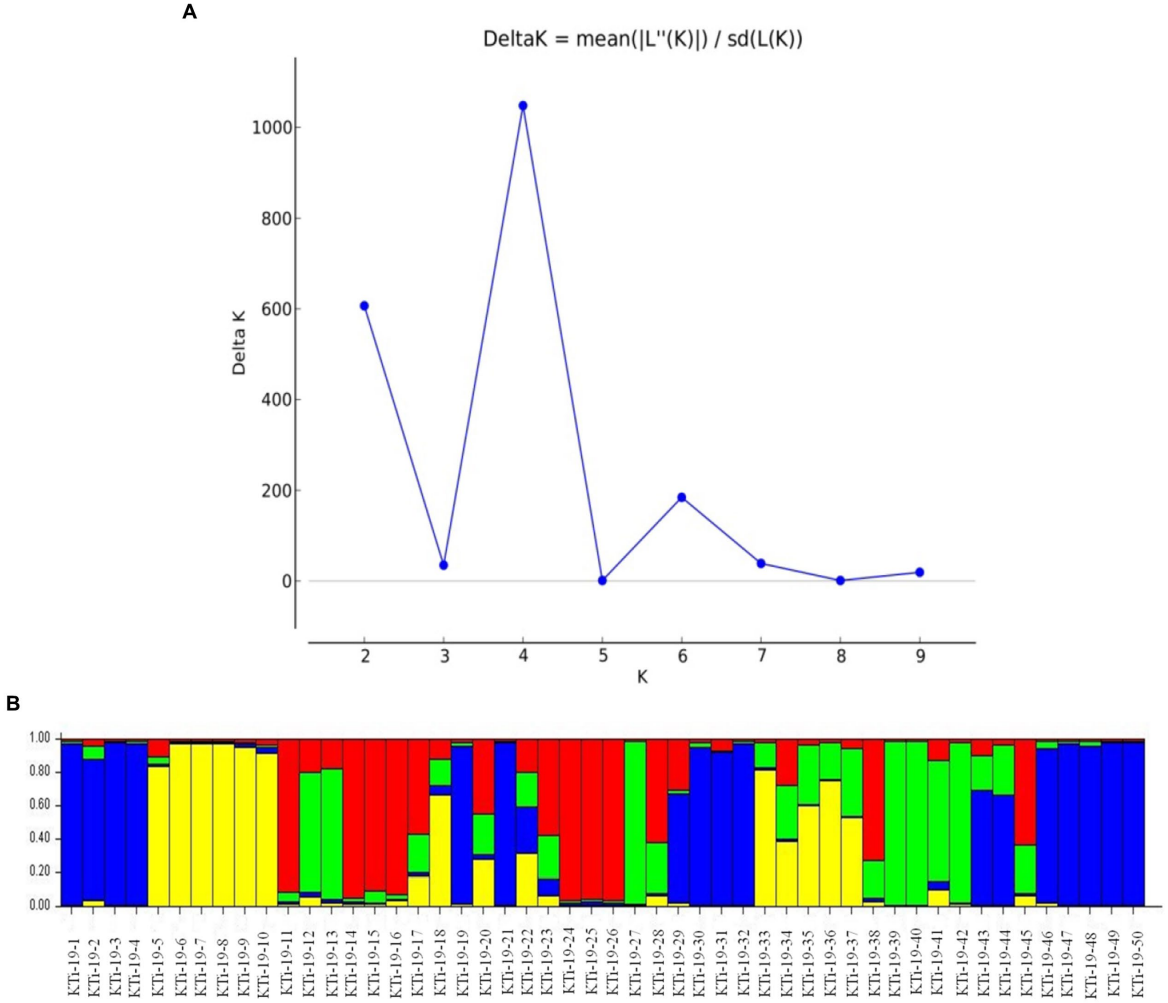
**(A)** ∆K values detected by novel polymorphic microsatellites using STRUCTURE HARVESTER software showing a clear delineation of four gene pools (*K* = 4) in 50 isolates of *Tilletia indica*; **(B)** Bar plot showing genetic structure of 50 *Tilletia indica* as revealed by STRUCTURE v2.3.3. The vertical coordinate of each subgroup indicates the membership coefficients for each isolate, and the numbers on the horizontal coordinate represent the isolates as mentioned in Table 1. Single color in each bar reveals the genetic background. Isolates with a mixture of more than one color indicate admixtures.

The ten polymorphic primer pairs identified in the current study resulted in the production of twenty-six different alleles, which were further deployed to estimate the genetic variability and kinship among different isolates of *T. indica*. The results of analysis of molecular variance (AMOVA) identified 94% genetic variation within population and 6% among population (Table 6). Further, it has been noticed that similarity coefficients values varied from 0.51 to 1.0 in all the isolates of *T. indica*. The dendrogram made at similarity index of ≥60% divided *T. indica* population into four major clusters (Figure 5). The Cluster-I occupied 15 isolates of *T. indica* (KTi-1, KTi-2, KTi-3, KTi-4, KTi-19, KTi-30, KTi-21, KTi-29, KTi-31, KTi-32, KTi-46, KTi-47, KTi-48, KTi-49, and KTi-50), while cluster II, III and IV included 2 (KTi-43 and KTi-44), 13 (KTi-5, KTi-37, KTi-6, KTi-7, KTi-8, KTi-9, KTi-33, KTi-34, KTi-35, KTi-36, KTi-10, KTi-18 and KTi-22) and 20 isolates (KTi-11, KTi-20, KTi-12, KTi-13, KTi-15, KTi-28, KTi-17, KTi-38, TiSSR45, KTi-14, KTi-16, KTi-23, KTi-24, KTi-25, KTi-26, KTi-27, KTi-41, KTi-42, KTi-39 and KTi-40) of *T. indica,* respectively. Similar results have been found with STRUCTURE program, when performed to assess similarity among different *T. indica* isolates at genetic level. The results of STRUCTURE analysis indicated a strong signal with a sole and clear peak at K = 4 (Figure 6) and further confirmed the prevalence of four genetically diverse groups in the studied population of *T. indica* representing seven Indian regions (Jammu, Himachal Pradesh, Punjab, Haryana, Uttarakhand, Uttar Pradesh and Rajasthan).

**Table 6:** Analysis of molecular variance (AMOVA) of *Tilletia indica* population.

Source	df	SS	MS	EV	%	ΦPT
Among Populations	6	77.557	12.926	0.599	6	0.064
Within Populations	43	378.143	8.794	8.794	94	
Total	49	455.700		9.393	100	

df, degree of freedom; SS, sum of squared observations; MS, mean of squared observations; EV, estimated variance; ΦPT, proportion of the total genetic variance among isolates within an population (*p* < 0.002).

## Discussion

Karnal bunt is one of the prime quarantine fungal threats to global wheat production and is reported to cause significant grain quality and economical loss. The most effective approach to dealing with KB disease is to breed disease-resistant wheat varieties, which demand a better and deeper understanding of the *T. indica* fungus at the genetic level (Bishnoi et al., 2020). In India, the North Western Plain Zone is the prime hot spot for KB disease, but only limited efforts have been made to decipher *T. indica* diversity at the genomic level. In this connection, current research attempts to make a comparative analysis of nine *T. indica* genomes available in the public domain for the development of novel and neutral microsatellite markers to dissect the genetic diversity and structure of the field population of *T. indica*. Earlier researchers have used a series of molecular markers or typing methods to analyze the genetic variability of *T. indica* (Avinash et al., 2000; Seneviratne et al., 2009; Aggarwal et al., 2010; Parveen et al., 2015; Aasma et al., 2022). Unfortunately, these methods are dominant types and are unable to establish analogous reproducibility of markers across populations at the genetic level, thereby being of limited significance, especially for comparative genotyping studies (Agarwal et al., 2008; Rao et al., 2018). In recent years, researchers have shown interest in exploring the potential of microsatellite markers in unzipping the genetic variation among fungal pathogens because of their ubiquitous nature, high polymorphism, co-dominance inheritance, and high level of allelic variation within the genome (Kumar et al., 2012; Mahfooz et al., 2012; Singh et al., 2014; Kashyap et al., 2015; Rai et al., 2016). Till date, the genomes of nine isolates of *T. indica* (DAOMC236416, DAOMC236414, DAOMC236408, PSWKBGH_1, PSWKBGD_1_3, PSWKBGH_2, RAKB_UP_1, TiK_1, and Tik) have been decoded, and information about them is available in the public domain (see Footnote 1). This gave us an opportunity to explore these genomes for microsatellite dynamics and prevalence. It is worth mentioning here that microsatellite sequences retrieved through bioinformatics and computational modes have similar utility when compared with microsatellites derived from genomic libraries. Additionally, the negligible expenditure of *in silico* mining and the high profusion of microsatellites in diverse types of genome sequences put this approach at the forefront of the discovery of novel microsatellite markers for population genomic studies. Therefore, nine genomes of *T. indica, viz.*, DAOMC236416, DAOMC236414, DAOMC236408, PSWKBGH_1, PSWKBGD_1_3, PSWKBGH_2, RAKB_UP_1, TiK_1, and Tik, were mined, and comparative analysis was done to know the distribution and dynamics pattern of microsatellite at whole genome level as well as to discover novel, neutral, and polymorphic microsatellite markers to get deep insight into the evolutionary relationship and dynamics of the *T. indica* population as well as for devising effective KB management strategies in wheat.

A wide spectrum of published research indicates that different taxa show distinct distribution patterns and dynamic microsatellite repeat motifs (Tautz et al., 1986; Toth et al., 2000; Wang et al., 2009). Likewise, in current research, the occurrence, abundance, and distribution of microsatellite motif repeats in nine genomes of *T. indica* of Canadian (e.g., DAOMC236416, DAOMC236414, and DAOMC236408) and Indian (PSWKBGH_1, PSWKBGD_1_3, PSWKBGH_2, RAKB_UP_1, TiK_1, and Tik) origin were mined. A series of research reports indicated a strong correlation between the size of the genome and microsatellite content (Karaoglu et al., 2005; Sahu et al., 2020). In contrast, no significant correlation was noticed between the total microsatellite content and the genome size in our study. Further, it was also observed that the RA of microsatellites did not uniformly exist in all nine genomes. Besides this, significant variation in the RA of each type of microsatellite motif was noticed in all the mined genomes. During comparative exploration of the *T. indica* genome, it was noticed that the RA and RD of microsatellites were at their maximum in the PSWKBGH_1 genome when compared with the other eight genomes. AG was a widely prevalent di-nucleotide motif repeat in all nine genomes of *T. indica*. Similarly, ACG/AGG was recorded as the most common tri-nucleotide motif in the genome. These observations were analogous to earlier reports where a high abundance of di-and tri-nucleotide motifs was noticed in the genomes of other organisms (Wang et al., 2009; Kumar et al., 2013; Sahu et al., 2020). We felt that these differences in densities and abundance of microsatellite motifs in *T. indica* could be due to the genomic organization of the isolates.

A series of published papers established the copious nature of tri-nucleotide repeats in contrast to other classes of motif in the coding regions of the genome (Kim et al., 2008; Mahfooz et al., 2012). Kashi and King (2006) mentioned that the dynamic mutations that happen in tri-nucleotide repeats influence diverse types of genetic functions. In the present research, efforts have been made to examine the microsatellite motifs presented in the *T. indica* genomes to get the real picture regarding the density of microsatellites in the different genomes of *T. indica*. The study confirmed the wide distribution of tri-nucleotide motifs in contrast to di-nucleotide motifs. Moreover, the trend of tri-nucleotide motif distribution showed conservancy across *T. indica* isolates. One feasible answer to these events could be selection against slippage mutations, which in turn might influence the stability and organization of the *T. indica* genome. It is worth mentioning here that the sequence composition of the motif type plays an important role in deciding the abundance of microsatellites in a genome. However, the sequence composition of the motif type did not illustrate conservancy across the species. The current research also established that (AG)n was the longest and most widely occurring microsatellite motif in the DAOMC236416 isolate, while in the cases of DAOMC236414, DAOMC236408, PSWKBGH_1, RAKB_UP_1, TiK_1, and Tik, (ACG)n was noticed as the most common microsatellite repeat unit. These findings also indicated that a sequence might harbor the most widely prevalent microsatellite motifs one or more times, but the total occurrence of the most frequent microsatellite motifs was different in *T. indica* isolates. Besides this, dissimilarity in the occurrence of polymorphic loci and the number of alleles per locus between genomic microsatellites is largely influenced by the origin of these sequences, owing to the fact that the coding region sequences are highly conserved in comparison to the non-coding region in a particular genome (Xie et al., 2018). Moreover, the size variation of alleles does not serve as a function of their repeating units. This indicates that insertions and deletions have a significant function in deciding the level of polymorphism in a genome. On parallel lines, the polymorphism pattern among the *T. indica* population composed of 50 different individuals has been studied. The study identified TiSSR27 and TiSSR17 as highly informative and neutral microsatellite markers for the genetic characterization of the *T. indica* population because of their high PIC values (0.50). The observed high level of polymorphism linked with microsatellites could be explained by replication slippage mechanisms responsible for creating SSR allelic diversity (Baird et al., 2010; Rai et al., 2016).

An ample of published literature indicates a significant amount of variation in the susceptibility of wheat cultivars to *T. indica*. This may be due to the high level of genetic variation among isolates with different characteristics for virulence and aggression (Mishra et al., 2001; Shakoor et al., 2015). It is important to mention here that the heterothallic nature of *T. indica* is the prime factor for generating continuous variation in the *T. indica* population (Fuentes-Davila and Duran, 1986; Thirumalaisamy et al., 2006). Hence, it becomes vital to distinguish *T. indica* isolates on the basis of aggressivity, which can be used for effective screening of germplasm against KB. Therefore, in the present investigation, efforts have been made to determine the aggressiveness of the 50 *T. indica* isolates on a set of three wheat cultivars, *viz.*, WL711, PBW343, and WH542. The results reflected a significant level of variation in the aggressivity of *T. indica* isolates. The study identified 24% of isolates as HA, while 30% and 46% of isolates were LA and MA, respectively. However, no strong correlation between the aggressivity and geographical origin of *T. indica* isolates and their genetic diversity was established. This means that *T. indica* isolates contain huge variation in terms of aggressivity in different wheat-growing sites in North India. Additionally, the occurrence of highly aggressive isolates of *T. indica* in Haryana, Rajasthan, Punjab, Uttar Pradesh, Uttarakhand, Jammu, and Himachal Pradesh also supported the movement of fungus from one region to another through seed or air. Similar results pertaining to the absence of region-specific virulence variability were also reported by Aasma et al. (2022).

Genetic variability and virulence potential of *T. indica* isolates were reported by earlier workers (Datta et al., 2000; Goates and Jackson, 2006; Parveen et al., 2013; Aasma et al., 2022). In the current study, DNA amplification with ten polymorphic microsatellite markers generated distinct amplicons that were therefore utilized as typing markers to characterize *T. indica* isolates derived from distinct geographical locations. These markers reveal the presence of a significant level of variation (94%) among the collected isolates at the genetic level. Although a flood of information pertaining to the assessment of the reaction of wheat germplasm to natural infection with KB is available (Kaur and Kaur, 2005; Riccioni et al., 2008; Aasma et al., 2022), limited research efforts were made to determine the variability of the large pool of *T. indica* isolates in terms of aggressiveness. Therefore, in the current study, a research plan was executed with the aim of capturing the real situation regarding the aggressivity of *T. indica* isolate prevalence in different wheat growing sites in the northern part of India. The outcome of the study clearly indicates robust genetic diversity among *T. indica* populations collected from seven Indian localities (Rajasthan, Haryana, Punjab, Uttar Pradesh, Uttarakhand, Jammu and Himachal Pradesh), where isolates of all three virulence categories existed. The significant effect of isolates and cultivars noticed in the current study further indicated that the pathogenic variation in *T. indica* isolates could be the outcome of gene-to-gene interaction dependent on isolate-host compassion, as documented by previous researchers (Datta et al., 2000; Ullah et al., 2012). However, an in-depth understanding of localized pathogenicity and genetic variability with a large pool of KB isolates is highly warranted with reported natural and novel polymorphic markers for developing sustainable and integrated modules for disease management and effective wheat breeding programs in the near future.

## Data availability statement

The datasets presented in this study can be found in online repositories. The names of the repository/repositories and accession number(s) can be found at: https://www.ncbi.nlm.nih.gov/, https://www.ncbi.nlm.nih.gov/genome/browse/#!/eukaryotes/8345/.

## Author contributions

The work was conceived and designed by PK and SK. The sampling survey was performed by PK, SK, PJ, and RK. Experiments were conducted by PK, RK, and AS. Field experiments, KB inoculations and data recording were conducted by PK, K, and SK. Bioinformatics and statistical data analysis was done by AK and K. The manuscript was drafted by PK. The final editing and proofing of manuscript was done by PJ, SK, and GS. All authors contributed to the article and approved the submitted version.

## Conflict of interest

The authors declare that the research was conducted in the absence of any commercial or financial relationships that could be construed as a potential conflict of interest.

## Publisher’s note

All claims expressed in this article are solely those of the authors and do not necessarily represent those of their affiliated organizations, or those of the publisher, the editors and the reviewers. Any product that may be evaluated in this article, or claim that may be made by its manufacturer, is not guaranteed or endorsed by the publisher.

## Footnotes

^1^https://www.ncbi.nlm.nih.gov/genome/browse/eukaryotes/8345/

^2^https://primer3.org/
