# Supplementary material for: Comparative analysis of nine Tilletia indica genomes for the development of novel microsatellite markers for genetic diversity and population structure analysis
Source: Front Microbiol. 2023 Jul 13;14:1227750. doi: 10.3389/fmicb.2023.1227750 (PMC10374028; doi:10.3389/fmicb.2023.1227750)
Supplement: Supplementary file 1 [file Data_Sheet_1.docx]

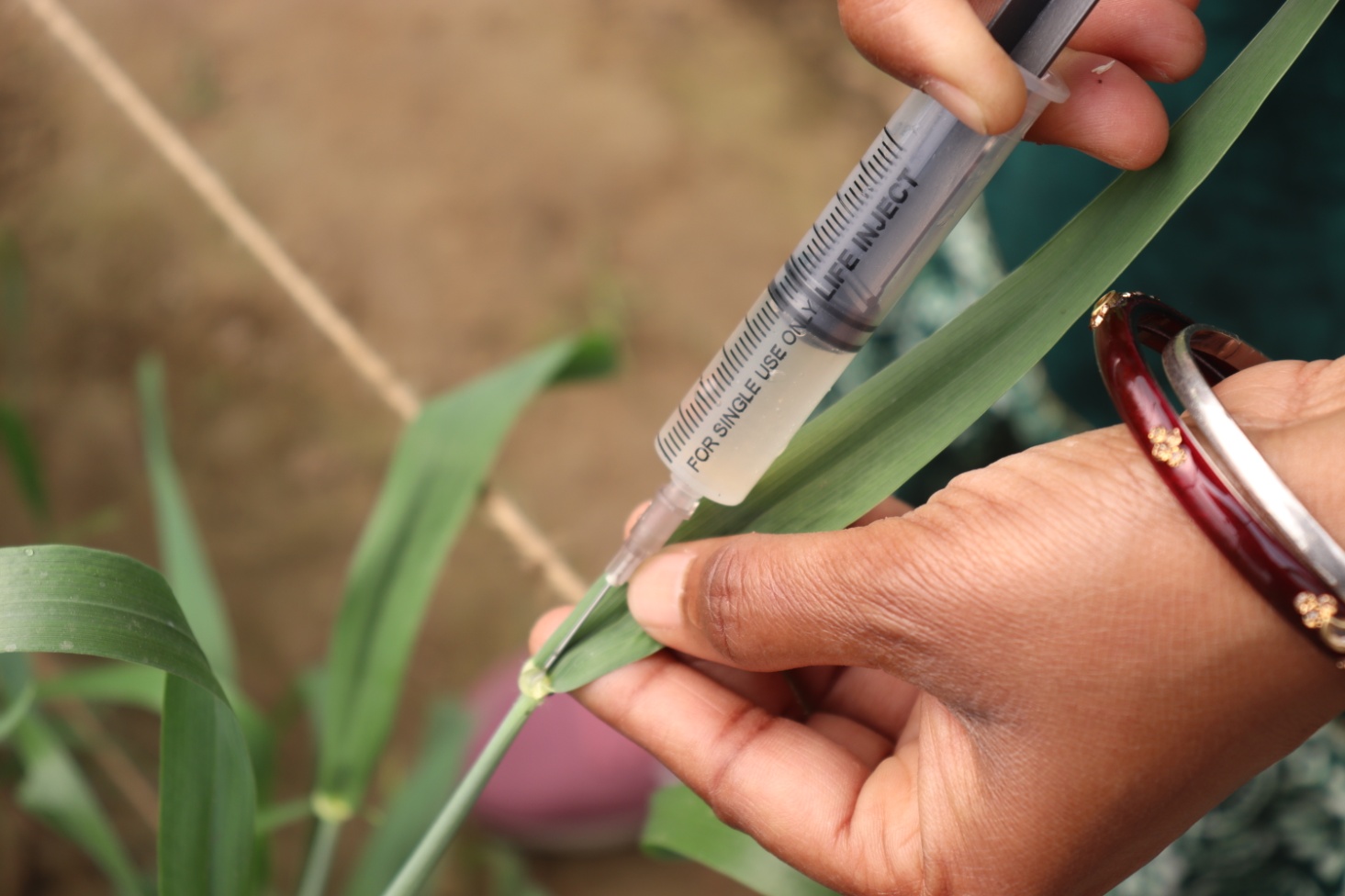


**Fig S1:** Field Inoculation with one millilitre of standardised liquid suspension of *T. indica* isolate in the ear-head with the help of a hypodermal syringe in single tiller at Zadock’s growth stage (ZGS 49, i.e., boot leaf stage)
